# Novel Framework for Treatment Response Evaluation Using PSMA PET/CT in Patients with Metastatic Castration-Resistant Prostate Cancer (RECIP 1.0): An International Multicenter Study

**DOI:** 10.2967/jnumed.121.263072

**Published:** 2022-11

**Authors:** Andrei Gafita, Isabel Rauscher, Manuel Weber, Boris Hadaschik, Hui Wang, Wesley R. Armstrong, Robert Tauber, Tristan R. Grogan, Johannes Czernin, Matthew B. Rettig, Ken Herrmann, Jeremie Calais, Wolfgang A. Weber, Matthias R. Benz, Wolfgang P. Fendler, Matthias Eiber

**Affiliations:** 1 Ahmanson Translational Theranostics Division, Department of Molecular and Medical Pharmacology, UCLA, Los Angeles, California;; 2 Department of Nuclear Medicine, Technical University Munich, Klinikum rechts der Isar, Munich, Germany;; 3 Department of Nuclear Medicine, University of Duisburg–Essen and German Cancer Consortium–University Hospital Essen, Essen, Germany;; 4 Department of Urology, University of Duisburg–Essen and German Cancer Consortium–University Hospital Essen, Essen, Germany;; 5 Department of Urology, Technical University Munich, Klinikum rechts der Isar, Munich, Germany;; 6 Department of Medicine Statistics Core, David Geffen School of Medicine, UCLA, Los Angeles, California; and; 7 Department of Urology, David Geffen School of Medicine, UCLA, Los Angeles, California

**Keywords:** metastatic castration-resistant prostate cancer, radionuclide treatment, PSMA PET, interim PET, ^177^Lu-PSMA

## Abstract

Our objective was to develop version 1.0 of a novel framework for response evaluation criteria in prostate-specific membrane antigen (PSMA) PET/CT (RECIP) and a composite response classification that combines responses by prostate-specific antigen (PSA) measurements and by RECIP 1.0 (PSA + RECIP). **Methods:** This was an international multicenter, retrospective study. One hundred twenty-four men with metastatic castration-specific prostate cancer (mCRPC) who underwent ^177^Lu-PSMA therapy and received PSMA PET/CT at baseline and at an interim time point of 12 wk were included. Pairs of baseline interim PET/CT scans were interpreted by consensus among 3 masked readers for appearance of new lesions. Tumor lesions were segmented, and total PSMA-positive tumor volume (PSMA-VOL) was obtained. Appearance of new lesions and changes in PSMA-VOL were combined to develop RECIP 1.0, which included classifications of complete response (RECIP-CR: absence of any PSMA-ligand uptake on interim PET/CT), partial response (RECIP-PR: decline ≥ 30% in PSMA-VOL and no appearance of new lesions), progressive disease (RECIP-PD: increase ≥ 20% in PSMA-VOL and appearance of new lesions), and stable disease (RECIP-SD: any condition but RECIP-PR or RECIP-PD). Changes in PSA levels at 12 wk by Prostate Cancer Working Group Criteria 3 were recorded. PSA + RECIP results were defined as response (PSA decline ≥ 50% or RECIP-PR/CR) or progression (PSA increase ≥ 25% or RECIP-PD). The study’s primary outcome measure was the prognostic value of RECIP 1.0 for overall survival (OS). The secondary outcome measure was the prognostic accuracy (C-index) of PSA + RECIP versus PSA responses. **Results:** Patients with RECIP-PD (*n* = 39; 8.3 mo) had a shorter OS than patients with stable disease (RECIP-SD) (*n* = 47; 13.1 mo; *P* < 0.001) or RECIP-PR (*n* = 38; 21.7 mo; *P* < 0.001). In identifying responders and progressors, PSA + RECIP had C-indices superior to those of PSA only: 0.65 versus 0.62 (*P* = 0.028) and 0.66 versus 0.63 (*P* = 0.044), respectively. **Conclusion:** PSMA PET/CT by RECIP 1.0 is prognostic for OS and can be used as a response biomarker to monitor early efficacy of ^177^Lu-PSMA in men with mCRPC. PSA + RECIP may be used as a novel composite endpoint in mCRPC clinical trial design.

## Visual Abstract

**Figure:**
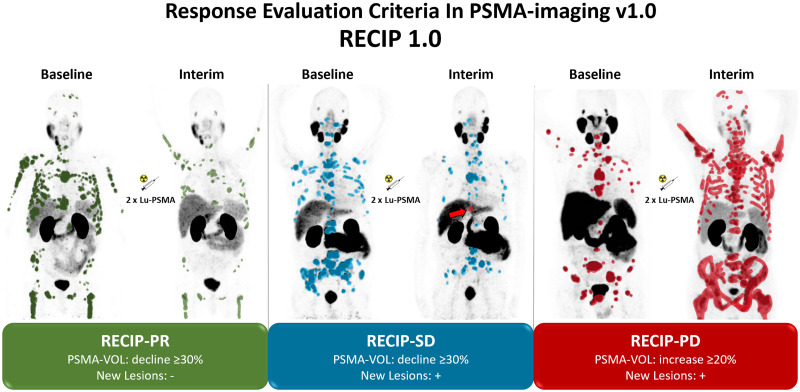

In metastatic prostate cancer, treatment response is typically evaluated using conventional imaging (CT and bone scanning) according to the Prostate Cancer Working Group Criteria 3 (PCWG3) guidelines. Prostate-specific membrane antigen (PSMA)–targeted PET/CT is a novel imaging technique that showed greater detection accuracy than conventional imaging in patients with high-risk primary prostate cancer (*1*). The U.S. Food and Drug Administration approved [^68^Ga]Ga-PSMA-11 PET/CT for different clinical settings in men with prostate cancer (*2*). However, there is little evidence for the prognostic value of PSMA PET/CT for response assessment in men with advanced prostate cancer (*3**,**4*). In our clinical experience using PSMA PET/CT for response evaluation of systemic metastatic castration-specific prostate cancer (mCRPC) treatments, a decrease in total disease burden can coincide with appearance of new lesions. This scenario, referred to as heterogeneous response, often leaves the treating physician in a clinical dilemma (*5*). Considering the rapidly evolving era of targeted treatments for mCRPC, accurate and early response assessment is urgently needed, but standardized response evaluation criteria for PSMA PET imaging have not been developed yet.

[^177^Lu]Lu-PSMA (^177^Lu-PSMA) is a small-molecule inhibitor that binds with high affinity to PSMA and delivers β-radiation. The randomized TheraP trial demonstrated superior prostate-specific antigen (PSA) responses and progression-free survival for ^177^Lu-PSMA-617 versus cabazitaxel (*6*). In the phase III VISION trial, ^177^Lu-PSMA-617 prolonged overall survival (OS) and imaging-based progression-free survival, when added to the standard of care in patients with metastatic castration-resistant prostate cancer (mCRPC) (*7*).

This study had 2 key objectives: first, to develop version 1.0 of a standardized framework for response evaluation criteria in PSMA PET/CT (RECIP) in men with mCRPC who undergo ^177^Lu-PSMA and, second, to develop a composite response classification that combines PSA measurements and PSMA PET/CT responses by RECIP 1.0 (PSA + RECIP).

## MATERIALS AND METHODS

### Patients and Study Design

In this international multicenter study, men with mCRPC treated with ^177^Lu-PSMA-I&T or ^177^Lu-PSMA-617 between December 10, 2014, and July 19, 2019, at the Technical University Munich, UCLA, and University Hospital Essen were retrospectively screened for inclusion. Eligible patients had received PSMA PET/CT at baseline (bPET) and after 2 cycles of treatment (interim PET/CT [iPET]), had received the same PET radiotracer at bPET and iPET, and had survival data available. ^177^Lu-PSMA was administered by intravenous injection of 6.0–8.5 GBq at 6- to 8-wk intervals. Treatment was continued up to a maximum of 4 or 6 cycles in the absence of progression and lack of severe toxicity according to the treating physician. bPET was performed within 10 wk before treatment. iPET was performed at 12 ± 2 wk after treatment initiation and 5 ± 1 wk after the second treatment cycle. Treatment protocols are detailed in the supplemental materials (available at http://jnm.snmjournals.org) (*8**–**13*). Serum PSA measurements were also collected at baseline and at 12 ± 2 wk. Changes in PSA levels at 12 wk relative to baseline were recorded and categorized according to PCWG3 criteria as response (≥50% decrease) or progression (≥25% increase) (*14*).

**FIGURE 1.**
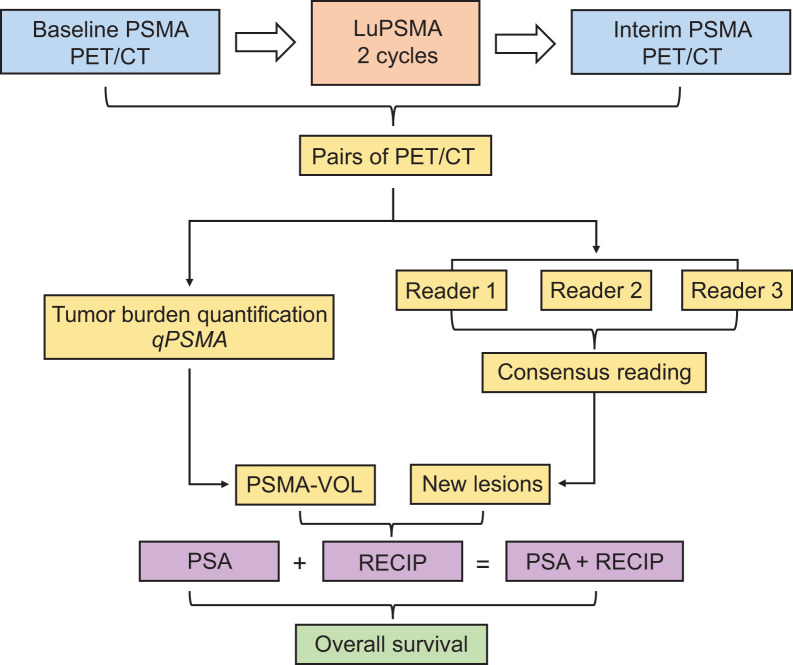
Study design. Patients who received bPET, were treated with at least 2 cycles of LuPSMA, subsequently received iPET, and had available survival data were included in this analysis. Tumor segmentation on both scans was performed using qPSMA software, and changes in total PSMA-VOL were calculated. Three independent readers interpreted scans for appearance of new lesion, and disagreement was solved by consensus reading. Changes in PSMA-VOL and consensus read results for appearance of new lesions were combined to develop RECIP. Serum PSA levels at bPET and at iPET were collected, and changes were recorded. PSA and RECIP responses were combined to develop composite response classification (PSA + RECIP). Prognostic ability for OS of PSA + RECIP vs. PSA only was tested.

The primary outcome measure was the prognostic value of RECIP 1.0 for OS. The secondary outcome measure was the prognostic ability of PSA + RECIP versus PSA only (Fig. 1).

All patients gave written informed consent to undergo clinical PSMA PET/CT. The retrospective analysis was approved by the Ethics Committees of each participating site (Technical University Munich, approval 115/18S; UCLA, approval 20-000954, University Hospital Essen, approval 19-8570-BO), and the committees waived the necessity for study-specific consent. Of note, the patient population in this study to develop RECIP was used to compare different criteria for response assessment in mCRPC (*15*).

### Imaging Acquisition

Images were obtained after application of PSMA ligands that were synthesized as described previously (*16**,**17*). Patients received an average (±SD) of 126 ± 4 and 317 ± 9 MBq of [^68^Ga]Ga-PSMA-11 and [^18^F]rhPSMA-7/7.3, respectively, via intravenous bolus. Image acquisition began 71 ± 6 min after tracer injection. Data from the CT scan were used for attenuation correction. Images were acquired using Siemens Biograph mCT (*n* = 115) and Siemens Biograph 64 (*n* = 9) scanners. All images were obtained in accordance with the European Association of Nuclear Medicine guidelines (E-PSMA) for treatment monitoring in patients with mCRPC, ensuring harmonized quantification (*18*). Standard vendor-provided image reconstructions were used. The institutional applied reconstruction parameters are summarized in Supplemental Table 1. Paired bPET and iPET were performed using the same PET/CT scanner and following same image reconstruction protocol.

### Image Analysis

PET/CT datasets from each participating site were anonymized and centralized.

#### Changes in Tumor Burden

**FIGURE 2.**
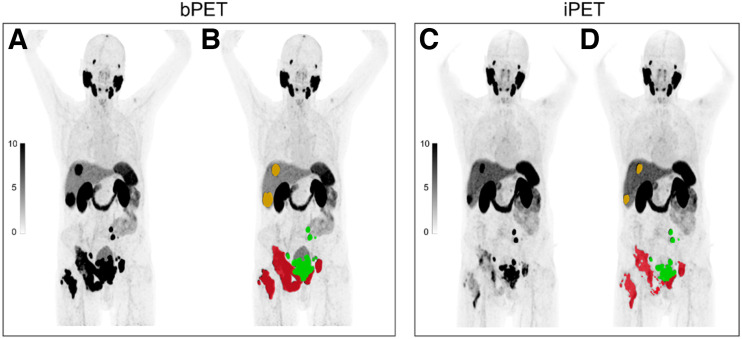
Changes in tumor burden on semiautomatic quantitative assessment of ^68^Ga-PSMA-11 PET/CT imaging using qPSMA software. Tumor lesions on bPET and iPET ^68^Ga-PSMA-11 PET/CT scans were segmented. Manual adjustments were performed when necessarily. Whole-body PSMA-VOL was extracted. DICOM images (A and C) are uploaded by user, and semiautomatic tumor segmentation (B and D) of bone (red), lymph node (green), and visceral (orange) metastases is obtained.

The PSMA-positive tumor lesions on bPET and iPET were annotated centrally by a nuclear medicine physician using the semiautomatic qPSMA software (Fig. 2). Segmentation workflow, time required for segmentation, and interuser reliability were described previously (*19*). The workflow for tumor segmentation is described in Supplemental Figure 1. The total PSMA-positive tumor volume (PSMA-VOL) was extracted. Percentage changes in PSMA-VOL on iPET relative to bPET were calculated. Cut points for categorization of partial response (PSMA-VOL_PR: 10%, 20%, 30%, 40%, and 50% decrease) and progressive disease (PSMA-VOL_PD: 10%, 20%, 30%, 40%, and 50% increase) were evaluated. The cutoff (%) with the highest prognostic accuracy for OS was further used to define PSMA-VOL_PR and PSMA-VOL_PD.

#### New Lesions

Pairs of bPET and iPET scans were read independently by 3 nuclear medicine physicians, who were masked to outcome data and were not involved in study design. Each reader was provided with full anonymized PET/CT datasets and was asked to assess the scans for new lesions following predefined criteria (Table 1). Disagreement among readers was solved in consensus sessions.

**TABLE 1:** Definitions of Criteria

Criterion	Definition
NL (new lesions)	Appearance of at least 1 new PSMA-positive lesion on iPET, which was defined as any new focal uptake of PSMA ligand higher than surrounding background, and each tumor SUV_max_ > mean SUV_mean_
RECIP	
RECIP-CR	Absence of any PSMA uptake on iPET
RECIP-PR	PSMA-VOL_PR without appearance of new lesions
RECIP-PD	PSMA-VOL_PD with appearance of new lesions
RECIP-SD	Insufficient decline in PSMA-VOL to qualify for PSMA-VOL_PR *or* PSMA-VOL_PR with appearance of new lesions *or* insufficient increase in PSMA-VOL to qualify for PSMA-VOL_PD *or* PSMA-VOL_PD without appearance of new lesions
Response classifications	
PSA	Response: ≥50% decrease; progression: ≥25% increase
RECIP	Response: RECIP-PR; progression: RECIP-PD
PSA + RECIP	Response: PSA ≥ 50% decrease or RECIP-PR/RECIP-CR; progression: PSA ≥ 25% increase or RECIP-PD

### Development of RECIP 1.0

Responses in PSMA-VOL were tested in conjunction with appearance of new lesions for associations with OS. We hypothesized, first, that patients with PSMA-VOL_PR without new lesions have OS superior to that of patients with PSMA-VOL_PR and new lesions and, second, that patients with PSMA-VOL_PD and new lesions have worse OS than patients with PSMA-VOL_PD without new lesions. On the basis of our hypothesis, RECIP 1.0 was developed and designed to classify patients into 4 categories: complete response (RECIP-CR), partial response (RECIP-PR), progressive disease (RECIP-PD), and stable disease (RECIP-SD) (Table 1). Associations of RECIP responses on iPET with OS were evaluated. Further, RECIP responses on iPET were combined with PSA responses at 12 wk to develop a novel composite response classification (PSA + RECIP). Definitions of all 3 response classifications are given in Table 1. The prognostic ability of PSA, RECIP, and PSA + RECIP responses for OS was evaluated.

### Statistical Analysis

Values are reported as average and SD or median and interquartile range (IQR) for continuous variables and as number and percentage for categoric variables. OS was estimated using the Kaplan–Meier method. The associations between OS and appearance of new lesions, changes in PSMA-VOL, and RECIP were evaluated using univariate Cox regression analyses. The hazard ratio (HR), its 95% CI, and the corresponding *P* values were derived. Appearance of new lesions and PSMA-VOL were tested separately and in combination to identify combined criteria with highest associations with OS. The prognostic ability of the PSA, RECIP, and PSA + RECIP classification systems was assessed using the Harrell concordance index (C-index) (*20*). Comparisons (*P* values) of C-indices were computed using the concordance function, which estimates the variance–covariance matrix between the correlated (repeated measure) C-indices (*21*). Agreement between readers in identifying new lesions on iPET was evaluated by Fleiss κ (KappaM package) (*22*). Analyses were performed using R software, version 3.4. A *P* value of less than 0.05 was considered statistically significant.

## RESULTS

From October 1, 2019, to December 18, 2019, retrospective data from 287 men with mCRPC were screened. Of these, 124 (43%) met the eligibility criteria and were included (Consolidated Standard of Reporting Trials diagram; Supplemental Fig. 2). One hundred fifteen (93%) of 124 patients were treated under compassionate-access programs, whereas 9 (7%) were enrolled in a phase II clinical trial (NCT03042312). Baseline characteristics are summarized in Table 2. Overall, 453 cycles of ^177^Lu-PSMA were administered, with a median of 4 cycles (IQR, 2–5). The median follow-up for survivors was 26.6 mo (IQR, 23.0–36.3 mo), and 113 (91%) of 124 patients were deceased at last follow-up. The cutoff for follow-up was August 19, 2020. The median OS was 13.5 mo (95% CI, 11.6–15.4 mo). Eighty-nine (72%) patients received [^68^Ga]Ga-PSMA-11 PET/CT, whereas 35 (28%) received [^18^F]PSMA-rh7/7.3 PET/CT. The median time between bPET and treatment initiation was 3.2 wk (IQR, 2.2–5.0 wk), whereas the median time between treatment initiation and iPET was 11.5 wk (IQR, 10.5–13.3 wk).

**TABLE 2:** Patient Characteristics (*n* = 124)

Characteristic	Data
Age (y)	73 (67–76)
Time since diagnosis of prostate cancer (y)	6 (4–11)
Gleason score at diagnosis*	
<8	36 (32%)
≥8	75 (68%)
M status at diagnosis	
M0	75 (60%)
M1	49 (40%)
Primary treatment	
Prostatectomy ± lymphadenectomy	70 (56%)
Local radiotherapy	12 (10%)
Systemic treatment	42 (34%)
PSA (ng/mL)	139 (37–427)
Lactate dehydrogenase (U/L)	286 (223–408)
Total alkaline phosphatase (U/L)	125 (81–250)
Hemoglobin (g/dL)	9.9 (11.3–12.7)
ECOG performance status	
0	31 (25%)
1	83 (67%)
2	10 (8%)
Previous mCRPC treatments	
Docetaxel	98 (79%)
Cabazitaxel	20 (16%)
Previous chemotherapy	99 (80%)
Abiraterone	111 (90%)
Enzalutamide	78 (63%)
Androgen-signaling-targeted inhibitors	123 (99%)
^ 223^Ra	24 (19%)
Prior lines of mCRPC systemic treatment	
1	9 (7%)
≥2	115 (93%)
≥3	71 (57%)
≥4	33 (27%)
Sites of disease on PSMA PET	
Bone	114 (92%)
Nodal	101 (81%)
Bone + nodal	92 (74%)
Visceral	32 (26%)
Bone + nodal + visceral	27 (22%)

* Data missing for 13 patients.

ECOG = Eastern Cooperative Oncology Group.

Qualitative data are number and percentage; continuous data are median and IQR.

### Associations of Appearance of New Lesion with Overall Survival

**FIGURE 3.**
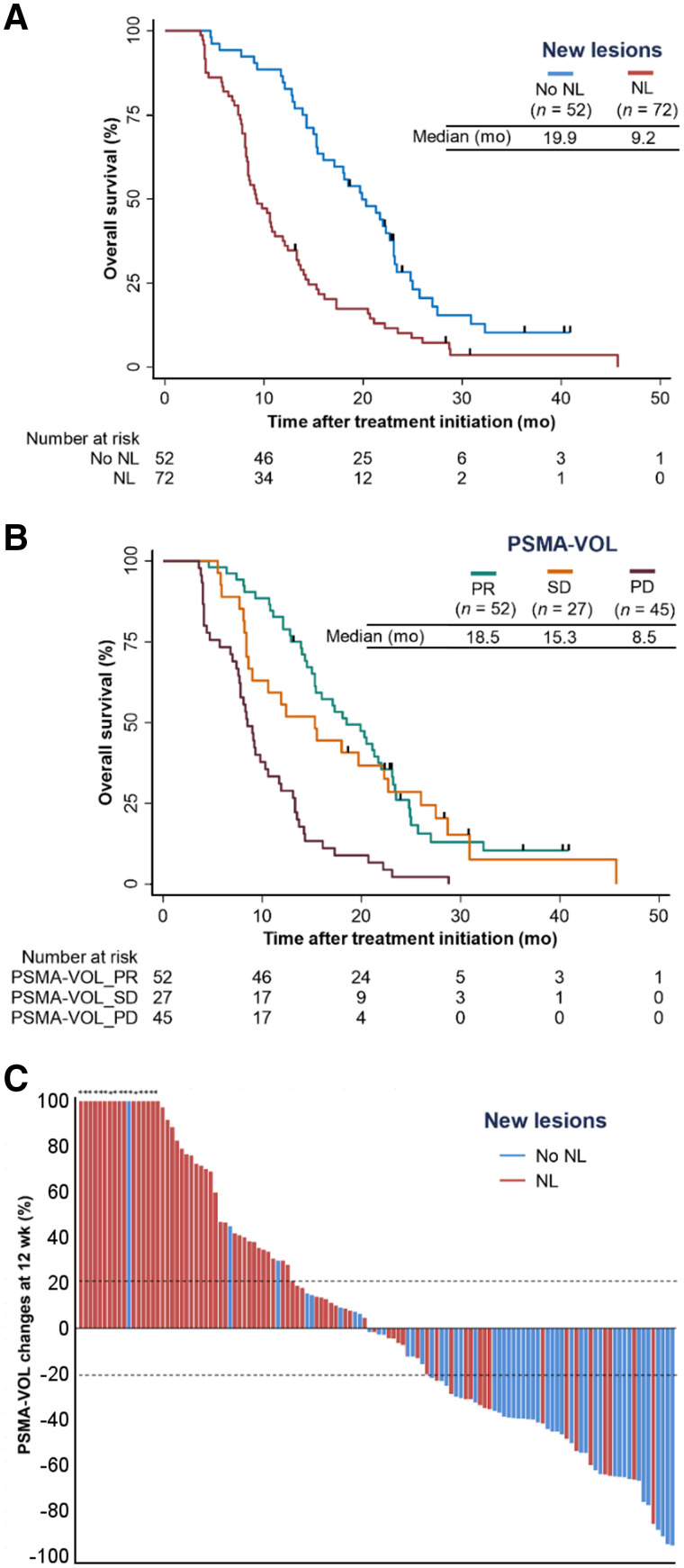
(A and B) Kaplan–Meier plots showing associations between appearance of new lesions (A) and response according to PSMA-VOL by qPSMA (B) with OS. Curves were truncated after 50 mo of follow-up because of low number of patients at risk. (C) Waterfall plot depicting relation between changes in PSMA-VOL and appearance of new lesions (NL) on PSMA PET. Asterisks indicate increase > 100% in PSMA-VOL changes.

On the basis of the consensus reads, 72 (58%) patients had at least 1 new lesion on iPET. Of these, 9 (13%) patients had new lesions on both PET and CT images. No new lesion was noticed on CT images only. Sixty-six (53%), 14 (11%), 24 (19%), and 12 (10%) of 124 patients had new bone, pelvic nodal, distant nodal, and visceral metastases, respectively. Appearance of at least 1 new lesion on iPET was associated with poor OS (HR, 2.32; 95% CI, 1.57–3.42; *P* < 0.001) (Fig. 3A). The results of independent reads in assessing new lesions are provided in Supplemental Figure 3. Substantial agreement among all 3 readers in identifying new lesions was noticed in 95 (77%) of 124 patients (κ = 0.69).

### Associations of Changes in Tumor Volume with Overall Survival

The median change in PSMA-VOL on iPET relative to bPET was −2.2% (IQR, −39.8 to +46.2). The C-indices for each cut point for definition of response and progression are provided in Supplemental Table 2. A cutoff of +20% had the highest prognostic value for PSMA-VOL_PD with OS (C-index, 0.64). Cutoffs of −20% and −30% had the highest but similar prognostic value for PSMA-VOL_PR with OS (C-index, 0.62), and the −30% cutoff was chosen to minimize the impact of measurement errors or biologic variability. Stable disease (PSMA-VOL_SD) was defined as either less than a 30% decrease or less than a 20% increase in PSMA-VOL.

OS was significantly superior in men with PSMA-VOL_PR (HR, 0.29; 95% CI, 0.19–0.45; *P* < 0.001) or PSMA-VOL_SD (HR, 0.35; 95% CI, 0.20–0.58; *P* < 0.001) compared with men with PSMA-VOL_PD (Fig. 3B). Sixteen (31%) of 52, 16 (59%) of 27, and 40 (89%) of 45 patients with PSMA-VOL_PR, PSMA-VOL_SD, and PSMA-VOL_PD, respectively, had appearance of new lesions on iPET (Fig. 3C).

### Establishment of RECIP and Associations with Overall Survival

#### RECIP-CR

Absence of any PSMA-ligand uptake on iPET was not observed.

#### RECIP-PR

Men with PSMA-VOL_PR and no evidence of new lesions had OS superior to that of men with PSMA-VOL_PR and appearance of new lesions (HR, 0.50; 95% CI, 0.25–0.93; *P* = 0.039). On this basis, the definition of RECIP-PR was maintained.

#### RECIP-PD

Men with PSMA-VOL_PD and appearance of new lesions had OS inferior to that of men with PSMA-VOL_PD but no evidence of new lesions (HR, 4.50; 95% CI, 1.36–14.90; *P* = 0.014) (Supplemental Fig. 4). On this basis, the definition of RECIP-PD was maintained. A case example of a patient with RECIP-SD is presented in Supplemental Figure 5.

**FIGURE 4.**
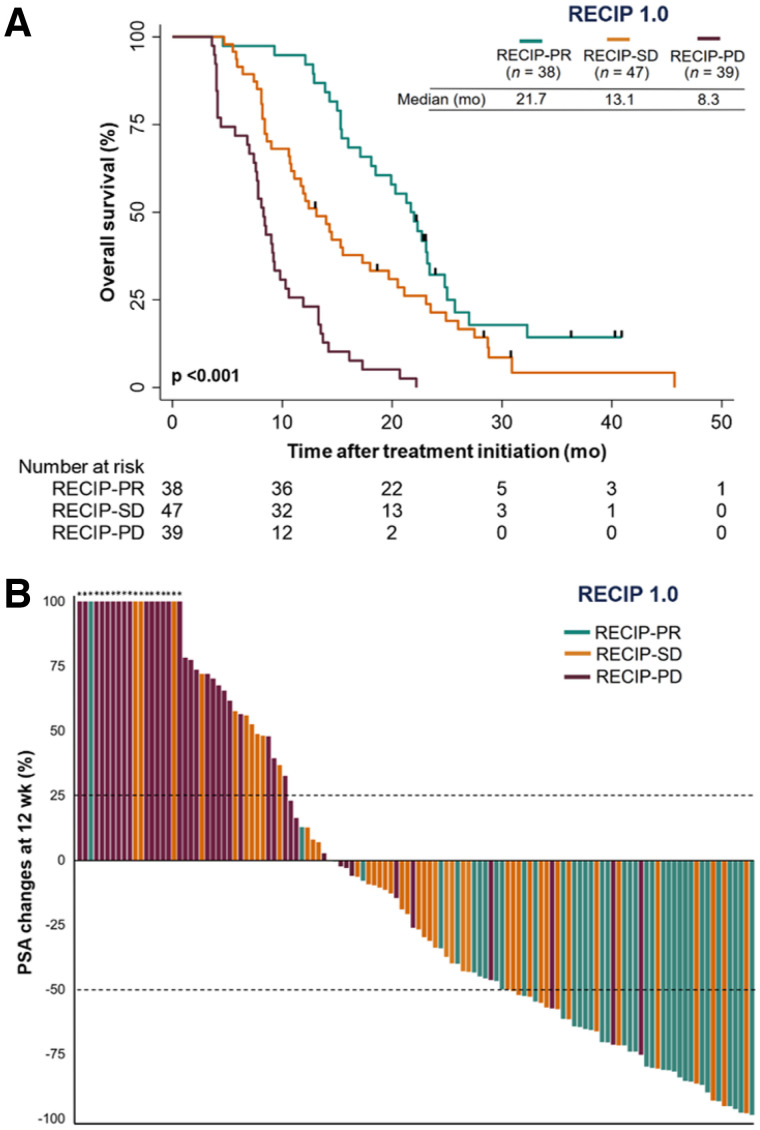
(A) Kaplan–Meier plot showing associations of imaging response according to RECIP with OS. Curves were truncated after 50 mo of follow-up because of low number of patients at risk. (B) Waterfall plot depicting relation between changes in PSA levels and imaging response according to RECIP. Asterisks indicate increase > 100% in PSA changes.

OS was superior in men with RECIP-PR (*n* = 38; HR, 0.17; 95% CI, 0.10–0.28; *P* < 0.001) or RECIP-SD (*n* = 47; HR, 0.30; 95% CI, 0.18–0.48; *P* < 0.001) compared with men with RECIP-PD (*n* = 39). RECIP-PR was associated with OS superior to that of RECIP-SD (HR, 0.56; 95% CI, 0.35–0.90; *P* = 0.017) (Fig. 4A). A waterfall plot displays the relationship between RECIP responses and PSA changes (Fig. 4B). The RECIP 1.0 classification method is summarized in Table 3.

**TABLE 3:** RECIP 1.0 Categories

Category	Criteria
RECIP-CR	Absence of any PSMA uptake on follow-up PET scan
RECIP-PR	>30% decrease in PSMA-VOL without appearance of new lesions
RECIP-PD	>20% increase in PSMA-VOL with appearance of new lesions
RECIP-SD	<30% decrease in PSMA-VOL with/without appearance of new lesions *or* ≥30% decrease in PSMA-VOL with appearance of new lesions *or* <20% increase in PSMA-VOL with/without appearance of new lesions *or* ≥20% increase in PSMA-VOL without appearance of new lesions

### Prognostic Ability of PSA, RECIP, and PSA + RECIP Classifications for Overall Survival

#### Response

**FIGURE 5.**
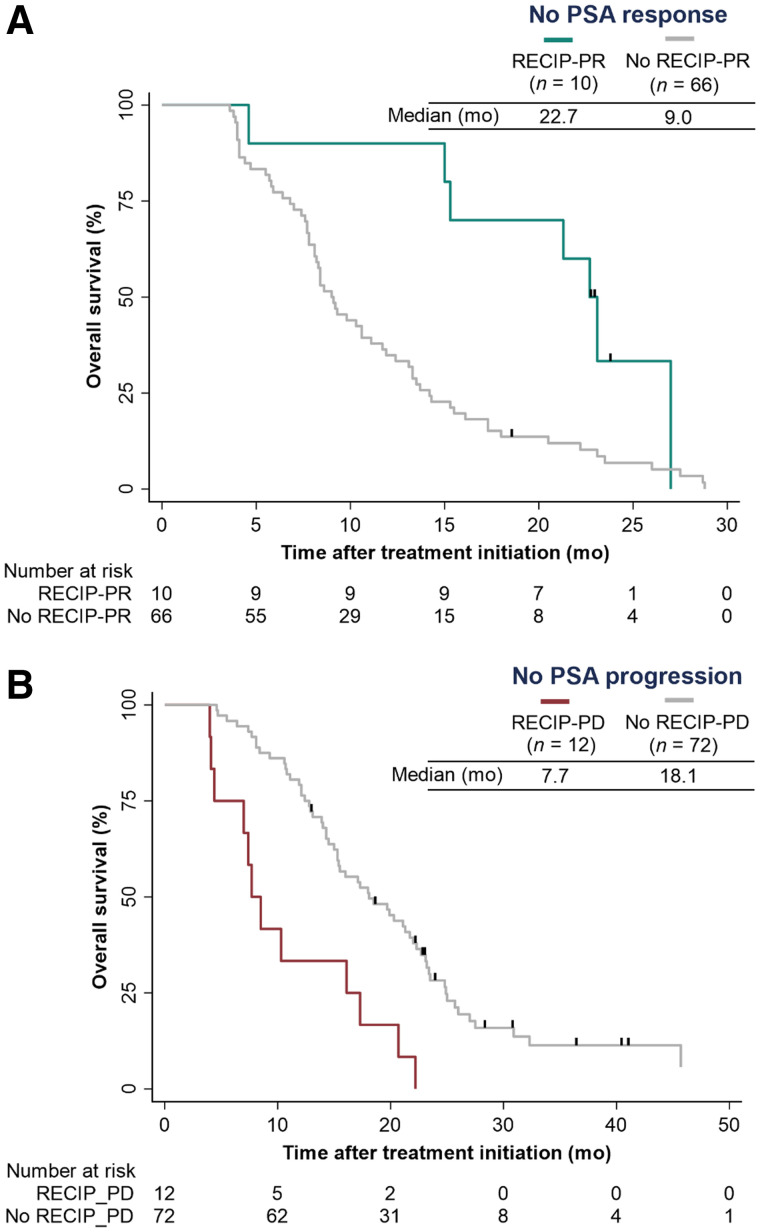
Kaplan–Meier plots showing associations with OS of response vs. nonresponse in PSMA PET/CT according to RECIP (RECIP-PR vs. no RECIP-PR) in patients without PSA response (A) and of progression vs. nonprogression in PSMA PET/CT according to RECIP (RECIP-PD vs. no RECIP-PD) in patients without PSA progression (B).

The C-index of response by PSA (0.63; 95% CI, 0.58–0.66) was similar to that by RECIP (0.63; 95% CI, 0.59–0.67; *P* = 0.830), and inferior to that by PSA + RECIP (0.66; 95% CI, 0.62–0.70; *P* = 0.028). Of 76 patients without a PSA response at 12 wk, 10 (13%) had RECIP-PR on iPET and had an OS superior to that without RECIP-PR (HR, 0.33; 95% CI, 0.15–0.73; *P* = 0.006; Fig. 5A).

#### Progression

The C-index of progression by PSA (0.62; 95% CI, 0.57–0.67) was similar to that by RECIP (0.65; 95% CI, 0.60–0.69; *P* = 0.210) and inferior to that by PSA + RECIP (0.65; 95% CI, 0.61–0.70; *P* = 0.044). Of 84 patients without PSA progression at 12 wk, 12 (14%) had RECIP-PD on iPET and had an OS inferior to that without RECIP-PD (HR, 3.33; 95% CI, 1.75–6.35; *P* < 0.001; Fig. 5B).

## DISCUSSION

Currently, the efficacy of ^177^Lu-PSMA and other systemic treatments of mCRPC is evaluated using conventional imaging (bone scanning + CT by PCWG3 criteria (*14*)), which may not accurately assess responses, especially for bone metastases, which are present in about 90% of mCRPC patients. PSMA PET/CT demonstrated a higher detection rate than conventional imaging (*1*); however, its prognostic role for treatment monitoring has not been established. Criteria for monitoring tumor response in PET imaging were described previously for ^18^F-FDG PET (PERCIST (*23*)), but PSMA PET and ^18^F-FDG PET image fundamentally different properties of the tumor tissue (PSMA expression and glucose metabolism, respectively). Thus, PERCIST is not applicable to PSMA PET imaging.

We developed RECIP 1.0 as the first—to our knowledge—evidence-based framework for response evaluation in prostate cancer using PSMA PET imaging. Two criteria have previously been proposed for the same purpose; however, these proposals included clinical information and were not based on multicenter validation (*24**,**25*). Compared with PERCIST, which uses measurements of individual lesions, RECIP 1.0 quantifies changes in total tumor volume, capturing the entire extent of disease. Binary PCWG3 classifies into progressive disease versus nonprogressive disease but lacks the ability to capture response by subcategorizing nonprogressive disease into complete response, partial response, or stable disease. Although identification of progressors may suffice in clinical practice, the objective response rate is commonly used in clinical trials as an endpoint to determining a drug’s efficacy (*26*). To enable assessment of the objective response rate of tumors, RECIP 1.0 was designed to distinguish true responders from patients with stable disease. A heterogeneous response by individual metastatic lesions is quite common during treatment of advanced mCRPC (*5*), as was confirmed in our patient population; that is, 13% of the patients had new lesions despite a response in tumor burden. These patients were classified by RECIP 1.0 as having stable disease and had a survival outcome different from true responders, who have no sign of progression (i.e., appearance of new lesions), and true progressors, who have both an increase in tumor burden and appearance of new lesions (median OS, 13.1 vs. 21.7 vs. 8.3 mo, respectively).

PSA response (≥50% decrease) is commonly used in phase II clinical trials of mCRPC as a primary endpoint to estimate antitumor activity. Our composite response classification system (PSA + RECIP) showed a prognostic accuracy for OS superior to that of PSA measurements only, highlighting the potential benefit of combining PSA and RECIP responses into a composite efficacy endpoint for clinical trials of mCRPC. The advantages of using composite endpoints include greater statistical precision and efficiency, that is, smaller sample sizes (which enable less costly trials and lower rates of treatment-related side effects) and a shorter follow-up (which enables faster availability of the results). Nevertheless, designing and implementing such endpoints can be challenging and hence require caution (*27*). In comparison to PSA measurements, PSMA PET/CT offers additional information about metastatic site and pattern of spread, as well as potential bone complications (e.g., spinal cord compression or fractures). This consideration is highly relevant to the clinical management of patients when adjuvant treatments can be considered, such as emergency surgery, radiation, or other metastasis-directed therapies.

Nomograms to predict outcome after ^177^Lu-PSMA using baseline patient and tumor characteristics were developed previously (*28*). The number of PSMA-positive metastases on pretherapeutic PSMA PET/CT was used as a surrogate marker of tumor volume for easier clinical implementation. Notably, the present analysis investigated dynamic changes in tumor burden during treatment. In this setting, changes in number of lesions are of limited use, and quantitative measurements of tumor burden are essential for accurate response evaluation.

Clinical use of PSMA PET/CT often lacks the ability to quantify whole-body disease burden because of high disease burden in metastatic settings. To enable quantitative assessment of total disease burden during treatment, different vendors are currently developing software tools. For this retrospective study, we used qPSMA for semiautomatic extraction of total tumor volume (*19*). It is in-house–developed and is freely available for widespread use. Other types of dedicated segmentation software might also become available to enable clinical implementation of PSMA PET/CT as a quantitative imaging biomarker in practice and trials (*29**–**31*). The prognostic value of iPET by RECIP is optimal (C-index, 0.65–0.70). The limited prognostic value of RECIP might be caused by an artificial decrease in PSMA expression because of dedifferentiation and not by a true decrease in tumor size.

The major limitations of this study were the lack of a prospective validation of RECIP criteria and an external validation of their threshold definition. Repeatability thresholds for tumor SUV measurements for ^68^Ga-PSMA-11 PET/CT were determined previously; however, tumor volumes were not included in the analysis (*32*). Another limitation of the study is that we could report only the prognostic, not the predictive, value of RECIP since we did not analyze data from a randomized trial powered for outcome. Future randomized studies monitoring tumor response with PSMA PET/CT are warranted to determine whether higher rates of PSMA response to a drug translate into a better clinical outcome. Also, this study could not compare the prognostic ability of RECIP versus PCWG3 criteria, since bone scans were not included in the clinical workup of ^177^Lu-PSMA radionuclide therapy at all institutions. Another limitation is that different PSMA PET radiotracers were used in this study, albeit consistent within patients. Further, the fact that the iPET was performed at 4–6 wk after the second cycle of treatment could impact both the ability to observe disease regression and the opportunity for new lesions to develop. Last, progression-free survival data were not included as a secondary endpoint because clinical assessment was not performed uniformly or at consistent time points across patients. Strengths of the study include the multicentric setting, a large patient population, and long-term follow-up survival data.

Our study has important clinical implications. First, it demonstrates the prognostic role of iPET as a response biomarker to monitor the efficacy of ^177^Lu-PSMA and possibly other mCRPC systemic therapies. After the positive outcome of the VISION registration trial (*7*), approval of ^177^Lu-PSMA is imminent. Early and accurate treatment response assessment by PSMA PET/CT may identify nonresponders early in the course of treatment and consequently decrease overtreatment and guide these patients to more effective therapies. Our interim time point of 12 wk for early response evaluation is in line with PCWG2 recommendations for mCRPC and with European Association of Nuclear Medicine procedure guidelines for ^177^Lu-PSMA therapy (*13**,**33*). End-treatment response evaluation using PSMA PET may also provide useful information on whether patients who complete the maximum number of ^177^Lu-PSMA cycles are candidates for a treatment rechallenge (*34*). However, only a subgroup of patients responds well and completes all cycles (i.e., 39/124 [31%] of our patients), and therefore, such analysis is limited by sample size. Further, there is currently no consensus among specialists on the maximum number of ^177^Lu-PSMA cycles. Second, RECIP 1.0 was developed as a potential powerful tool to determine imaging responses and to better assess heterogeneous response, and third, our findings suggest the value of adding PSMA PET/CT imaging to PSA measurements in evaluating treatment efficacy, which may result in higher precision and patient outcome of mCRPC trials.

## CONCLUSION

RECIP 1.0 was developed as an evidence-based novel framework to assess tumor response early in the course of treatment in mCRPC using PSMA PET/CT. PSA + RECIP is proposed as a novel composite-efficacy endpoint for clinical trials of mCRPC. PSMA PET/CT can be used as a response biomarker for early monitoring of the efficacy of ^177^Lu-PSMA and potentially other mCRPC treatments. Validation of the findings in a prospective setting is warranted.

## DISCLOSURE

This study was partly funded by the Prostate Cancer Foundation (grant 21YOUN18). Andrei Gafita is supported by the Prostate Cancer Foundation (grant 21YOUN18), a UCLA Jonsson Comprehensive Cancer Center fellowship award, and a Dr. Christiaan Schiepers postdoctoral fellowship award. Boris Hadaschik received financial support from the German Research Foundation (DFG HA 5160/5-1). Jeremie Calais is supported by the Prostate Cancer Foundation (grant 20YOUN05 and 19CHAL02) and the Society of Nuclear Medicine and Molecular Imaging (2019 Molecular Imaging Research Grant for Junior Academic Faculty). Wolfgang Fendler received financial support from the German Research Foundation (Deutsche Forschungsgemeinschaft, DFG, grant FE1573/3-1/659216), Mercator Research Center Ruhr (MERCUR, An-2019-0001), IFORES (D/107-81260, D/107-30240), Doktor Robert Pfleger-Stiftung, and Wiedenfeld-Stiftung/Stiftung Krebsforschung Duisburg. Hui Wang received financial support from the China Scholarship Council (CSC). Matthias Eiber reports prior consulting activities for Blue Earth Diagnostics Ltd., Novartis, Telix, Progenics, Bayer, Point Biopharma, and Janssen and a patent application for rhPSMA. Boris Hadaschik reports personal fees and nonfinancial support from Bayer, BMS, AstraZeneca, Lightpoint Medical, and Janssen and personal fees from Pfizer and ABX outside the submitted work. Jeremie Calais reports prior consulting activities outside the submitted work for Advanced Accelerator Applications, Blue Earth Diagnostics, Curium Pharma, GE Healthcare, IBA Radiopharma, Janssen Pharmaceuticals, POINT Biopharma, Progenics Pharmaceuticals, Radiomedix, and Telix Pharmaceuticals. Johannes Czernin is a founder of, board member of, and holds equity in Sofie Biosciences and Trethera Therapeutics and was a consultant for Endocyte Inc. (VISION trial steering committee), Actinium Pharmaceuticals, and Point Biopharma, outside the submitted work. Intellectual property is patented by the University of California and licensed to Sofie Biosciences and Trethera Therapeutics. Ken Herrmann reports personal fees from Bayer, Sofie Bioscience, SIRTEX, Adacap, Curium, Endocyte, BTG, IPSEN, Siemens Healthineers, GE Healthcare, Amgen, Novartis, ymabs, Bain Capital, and MPM Capital; other fees from Sofie Biosciences; nonfinancial support from ABX; and grants from BTG, outside the submitted work. Wolfgang Fendler reports fees from BTG (consultant), Calyx (consultant), RadioMedix (image review), Bayer (speakers bureau), and Parexel (image review), outside the submitted work. Wolfgang Weber was on advisory boards and received compensation from Bayer, Blue Earth Diagnostics, Endocyte, ITM, RayzeBio, and Pentixapharm and has received research support from BMS, Imaginab, Ipsen, and Piramal. No other potential conflict of interest relevant to this article was reported.

## Notes

KEY POINTS **QUESTION:** Can a framework to evaluate the efficacy of ^177^Lu-PSMA using PSMA PET/CT imaging be developed? **PERTINENT FINDINGS:** The RECIP 1.0 classification method has been developed and found to have prognostic value for OS. **IMPLICATIONS FOR PATIENT CARE:** RECIP 1.0 can assess tumor response in mCRPC patients using PSMA PET/CT.
